# Perception of Personal Participation of the Nurses in Resuscitation Procedures: A Qualitative Study

**DOI:** 10.3390/medicina60020196

**Published:** 2024-01-24

**Authors:** Anton Koželj, Maja Strauss, Vita Poštuvan, Anže Strauss Koželj, Matej Strnad

**Affiliations:** 1 Faculty of Health Sciences, University of Maribor, 2000 Maribor, Slovenia; maja.strauss@um.si; 2 Slovene Center for Suicide Research, Andrej Marušič Institute, University of Primorska, 6000 Koper, Slovenia; vita.postuvan@upr.si; 3 III Gimnazija Maribor, Gosposvetska Cesta 4, 2000 Maribor, Slovenia; anze.strauss-kozelj@tretja.si; 4 Emergency Department, University Clinical Centre Maribor, 2000 Maribor, Slovenia; matej.strnad@um.si; 5 Center for Emergency Medicine, Prehospital Unit, Community Healthcare Center, 2000 Maribor, Slovenia; 6 Faculty of Medicine, University of Maribor, 2000 Maribor, Slovenia

**Keywords:** resuscitation, personal experiences, paramedics, prehospital environment

## Abstract

*Background and Objectives:* Resuscitation is one of the most stressful tasks in emergency medicine. The participation of nurses in this procedure can have specific effects on them. In this research, we wanted to find out what these effects are. *Materials and Methods:* A qualitative approach by conducting semi-structured interviews was used, and a thematic data analysis of the recorded interviews was carried out. The collected data were transcribed verbatim, with no corrections to the audio recordings. The computer program ATLAS.ti 22 was used for the qualitative data analysis. *Results:* Eleven male registered nurses were interviewed, with an average of 18.5 years of experience working in a prehospital environment (max. 32/min. 9). A total of 404 min of recordings were analyzed, and 789 codes were found, which were combined into 36 patterns and 11 themes. As the most stressful situations, the interviewees pointed out the resuscitation of a child, familiar persons, conflicts with the environment, conflicts within the resuscitation team, nonfunctioning or insufficient equipment, complications during resuscitation, and resuscitating a person only for training. As positive effects, the interviewees cited successful resuscitations or their awareness that, despite an unsuccessful resuscitation, they did everything they could. *Conclusions:* Participation in these interventions has a specific positive or negative impact on the performers. The interviewees shared the opinion that they can cope effectively with the adverse or stressful effects of resuscitation. Yet, despite everything, they allow the possibility of subconscious influences of this intervention on themselves.

## 1. Introduction

Cardiac arrest presents one of the most stressful situations in emergency medical care. In such situations, action must be immediate and entirely appropriate; otherwise, a person with cardiac arrest has no chance of survival [1,2,3]. Cardiac arrest is the third leading cause of death in Europe [4]. The factors associated with survival are the initial rhythm, location of the cardiac arrest, and presence of monitoring at the time of the cardiac arrest [5]. The survival of persons resuscitated outside the hospital is usually poor. An essential factor is the eyewitnesses’ early implementation of basic resuscitation procedures [6,7,8].

In the case of sudden cardiac arrest in the hospital, the resuscitation team is immediately activated. For the above reasons, the medical personnel at the event are under tremendous pressure. Without a doctor, a nurse must manage resuscitation [9,10]. In the prehospital environment, however, the availability of a doctor is often limited, so all decisions and the implementation of resuscitation must be fully taken over by other medical professionals present on the spot (paramedics, nurses, etc.). Uncertain circumstances, insufficient information about the person’s condition, a small number of available personnel, equipment, additional help, the presence of relatives, etc., all pose additional stress when making decisions. Decisions (including initiating, abandoning, or terminating resuscitation) must be made immediately. In Slovenia, for a pre-expressed will of DNR (do not resuscitate), the patient must obtain the consent of the personal physician and the regional guardian of the patient’s rights, who separately explain his rights to him and judge that the patient can make decisions for themself to make this kind of decision. The accepted decision must then be jointly signed and notarized. The person must always carry this form with him or hand it over to the healthcare workers (or relatives who do it for him), since in our country, this kind of decision cannot be entered into a central health information system. In a hospital environment, the decision to not resuscitate a patient can be made by two specialist doctors, one of whom is not the patient’s attending physician. The decision is recorded in the patient’s medical record.

It is estimated that 40–84% of all resuscitation attempts result in the immediate or imminent death of the patient within 24 h. Such a large-scale failure can leave healthcare workers with unpleasant psychological consequences [11,12]. The stress caused by unsuccessful resuscitations among nurses has already been researched by Cole and his colleagues, who named this “post-code stress”, which results from a conflicting relationship between the expected and actual outcome of the resuscitation. He and his colleagues believe that the participation of nurses in resuscitation procedures could create a uniquely increased level of psychological stress [13]. Paramedics and nurses who work in emergency medical care units may experience post traumatic stress disorder (PTSD), which may be caused by being present or participating in resuscitation, sharing a challenging event, or long-term exposure to stress [14,15,16]. Among hospital nursing staff, emergency and psychiatric nurses are reported to have the highest prevalence of PTSD [17,18]. It is possible to observe a higher incidence of PTSD among paramedics than among the general population [19,20]. The incidence of PTSD within different professional intervention groups (paramedics, firefighters, and police officers) varies between 0% and 46% [21,22]. Paramedics, however, have a higher prevalence of developing PTSD than firefighters and police officers [19,23]. This may result from work requirements and specifically, more challenging events encountered by employees in the field of emergency medicine. Resuscitation is one of them.

In Slovenia, we do not have a paramedic level of education as is known in many other countries. Nurses and emergency medical technicians with additional knowledge work in prehospital areas. According to the regulations on emergency prehospital medical services in Slovenia, two types of teams operate in the prehospital environment. The first is the mobile reanimobile unit, which consists of three members, one of whom is a doctor, the second is a registered nurse, and the third is an emergency medical technician. The second is the mobile emergency ambulance unit, which consists of two members: a registered nurse and an emergency medical technician. The nurses involved in our research alternate in both positions. When working without a doctor, they are limited in many procedures that they may only perform with a doctor’s order. However, the aforementioned does not cover the field of resuscitation, which can be completed independently. 

The purpose of the study was to obtain data on the subjective experiences of nurses’ feelings when offering help to a person in sudden cardiac arrest.

## 2. Materials and Methods

### 2.1. Sample Selection

For this research, we used a qualitative research approach using a thematic analysis of the collected data. We conducted 11 interviews among typical representatives of a specific population of nurses working in the prehospital environment. With this number, we have reached data saturation. With new interviews, we check the characteristics of the created concepts and categories. By carrying out the last two interviews, we did not obtain any new data, with the help of which we could come to new insights. The interviewed individuals were selected according to the “good informant” criterion, which means that we included persons with good knowledge of the research topic, adequate ability to reflect, and the ability to participate in interviews [24]. The criteria for selecting interview candidates were several years of work experience working in a prehospital environment, successful teaching work (e.g., international instructorship of ALS), and a certain level of recognition and respect in the professional environment in Slovenia. The selection was made based on the researcher’s personal knowledge or the recommendation of superiors. We then agreed on the date and time of the interviews with the selected persons. None of the respondents refused to participate in the research. Geographically, we chose the interviewed persons from eight different medical institutions, which were evenly spread across Slovenia. The interviews were arranged with the interviewees and conducted in a relaxed and calm environment (office, home environment, etc.), without distractions, through a personal conversation between the researcher and the interviewee. The person with the longest experience had 32 years of work experience in emergency medicine (prehospital care). In comparison, the person with the least years of work experience in emergency medicine (prehospital care) had been working for 9 years. On average, the interviewees had 18.5 years of experience (prehospital care). 

### 2.2. Selection of Interview Questions

The interview was semi-structured. The interviewees answered the questions, and the conversation expanded beyond the set framework. The interview questions were formulated based on a literature review, the data obtained from a quantitative survey, and two pilot interviews. Furthermore, the researchers had previously consulted two experts in this field about the appropriateness of the questions. 

Indicative interview questions were as follows: Does taking part in resuscitations generally put a strain on you? What kind of stresses do you face (physical and psychological) during resuscitations? Do you feel that you encounter any ethical dilemmas or doubts when resuscitating? How do you experience resuscitation abandonments? When to stop resuscitation procedures? How do you decide about ending resuscitation (e.g., the patient has not recovered vital signs)? How does this affect you? How does the age of the resuscitated person affect you (child/elderly)? How do you assess your knowledge of performing resuscitation (TPO and DPO)? What is your opinion on your competencies during resuscitation performed without a doctor present? Do you often find yourself in a situation where you disagree with the decisions of the team leader (doctor) or other resuscitation participants? How do you react, and how does it affect you? What is your opinion about the decision to DNR (do not resuscitate)? What is your opinion about the presence of relatives during resuscitation, and how do you experience such situations? How often do you encounter conflict before, during, and after resuscitation procedures? How do you resolve them, and how do they affect you? How are you affected by circumstances in the environment that may put you at risk as a provider? What disturbs you most during resuscitations? What concerns you most in resuscitations? When does participation in resuscitation procedures have a positive impact on you? Do you think that taking part in resuscitation procedures affects your professional or private life to some extent? Have you considered changing your job or even your profession due to your participation in resuscitation procedures? Would you like to have more conversations after resuscitation procedures? Who would you like to talk to about it (colleagues, supervisors, psychologists, etc.)? Do you accept the possibility that participation in such procedures may have impacted you, but you are currently not aware of it? Would you like further training, and if so, in which areas? Do you still find resuscitation a professional and personal challenge? 

### 2.3. Data Analysis

The subject of the analysis was the interview transcripts recorded during the interviews. In a short time interval after the implementation, one of the researchers listened to them several times and made the first notes about the obtained data. A thematic data analysis was used to analyze the data obtained in the interviews. In interpreting the data obtained from the interviews, we used the approach of Sundler, who, together with her colleagues, provided guidelines for conducting a thematic analysis based on descriptive phenomenology [25]. In the introduction, we tried to find different meanings and their complexities by reading the data. This approach also includes the personal involvement of the researcher in the data analysis. The data analysis continues by forming meanings and themes, later organized into meaningful wholeness. Two researchers (coauthors of the article) separately approached the analysis of the transcribed interviews (multiple readings, searching for meanings, patterns, and themes). After a one-month break, we reflected on the collected data and corrected them. Upon completing the analysis, the researcher presented the data to one of the interviewees and checked the researcher’s understanding of the obtained data. A typist transcribed the interviews in the researcher’s presence (joint listening of audio recordings of the interviews). The interviews were transcribed verbatim, and we made no corrections to the audio recordings. The data analysis was performed manually while reading texts, marking codes and meanings, and writing notes. Later, we used the ATLAS.ti 22 qualitative data analysis computer program for the final analysis. 

## 3. Results

The quotations represent a direct transcript of the participants’ interviews. The citations are coded based on content matching with the code. The codes, therefore, represent meanings that are combined into semantic patterns. We conducted a total of 404 min of interviews. The longest interview lasted 48 min, and the shortest 25 min. The average time interval of the interview was 36.7 min. When analyzing the qualitative data, we determined 789 codes based on the statements, from which we then formed semantic patterns. Thus, we defined 36 meaning patterns, from which we finally created 11 themes (Table 1). 

The presentation of 36 semantic patterns is as follows: 1. CPR affects me; 2. Error warning—I warn others; 3. Error alert—others alert me; 4. Professional attitude; 5. Education; 6. Additional conversations; 7. Self-analysis; 8. Psychosomatic response; 9. Stressful situation; 10. Presence of relatives: YES/NO; 11. Time for yourself; 12. Ethical dilemmas; 13. Personal satisfaction; 14. Personal growth; 15. Personal feelings: respect for health professionals/disrespect for health professionals; 16. Unconscious effects of resuscitation on me: YES/NO; 17. Competencies and powers; 18. Vices: YES/NO; 19. Danger; 20. Attitude towards DNR (do not resuscitate): I support DNR/I do not support DNR; 21. Attitude towards organ donation: I support/I do not support; 22. Resuscitation according to the age of the person being revived: (22a) resuscitation of a child; (22b) resuscitation of an elderly person; 23. Powerlessness; 24. Previous experiences: you associate resuscitation with something familiar; 25. Physical efforts during resuscitation; 26. Mental stress during resuscitation; 27. Positive effects of resuscitation on practitioners; 28. The difference in resuscitation in the prehospital or hospital environment; 29. Complications during resuscitation; 30. Making decisions; 31. “Debriefing”—conversations after resuscitation; 32. Professional challenge; 33. Resuscitation of a familiar person; 34. Knowledge of CPR; 35. Conflicts during resuscitation; 36. Teamwork.

### 3.1. Presentation of 11 Designed Themes with an Indication of the Most Characteristic Quotes

The following chapter presents the 11 designed themes and the most characteristic statements regarding the topic. The quotations have been translated from Slovenian to English to preserve their originality as much as possible.

#### 3.1.1. Effects of Resuscitation on Performers’ Emotions

In the first topic, we found that participation in such procedures affects the emotions of the interviewed persons. We defined both positive and negative emotional responses. Positive emotional reactions include being present during successful resuscitations, especially those that ended with the ROSC (return of spontaneous circulation) and the reanimated person’s discharge from the hospital with a relatively good neurological outcome. Several interviewees indicated very positive feelings about the resuscitated person’s gratitude. A more significant emotional response results from the resuscitation of children or a resuscitation that the respondents can relate to some familiar matter from their lives (resuscitations that evoke certain associations or can be connected to familiar persons). As factors that have a negative effect on their emotions, the interviewees mentioned resuscitation only for the sake of practice, the inadequate operation of resuscitation equipment or the lack thereof, disagreement with team members or team leader, unsuccessful resuscitation (nevertheless, this can still have positive effects when considering that they did everything in their power), and the feeling of helplessness. It was expressed as they had done everything in their power, yet the desired results were not achieved.

Characteristic quotes: 

I:9 “You associate resuscitation with something familiar. There is also a difference if you are resuscitating a child and, at the same time, you have a child of approximately the same age at home.”

I:2 “That many times the problem was that we still went to perform resuscitation, solely because of the relatives, because the relatives were there and crying and it was bad for them...”

I:4 “Some members of the team may be a little too driven and refuse to give up on the resuscitation itself.”

I:5 “Yes, it is often clear that the resuscitation will be unsuccessful, and then a feeling of helplessness washes over you, which is not pleasant.”

#### 3.1.2. Professional Attitude

Regarding the second theme, we found that the interviewees believed they acted professionally during resuscitation. They are confident they have good knowledge and skills to perform resuscitations. For most people, resuscitation still presents a professional challenge because, as one interviewee stated, “every resuscitation is different”. They highlighted available guidelines for resuscitation (adult and pediatric basic and advanced life support guidelines from the European Resuscitation Council) for standardized work. Most interviewees stated that it is easier for them to carry out resuscitation in the presence of a doctor since the burden of the decision is on the doctor as the team leader. At the same time, they (especially those from smaller units) highlighted that they sometimes work with a doctor who does not follow the current guidelines. This can mean facing a stressful situation and a possible conflict or dilemma for them. 

Characteristic quotes: 

I:1 ”Reanimation is not that hard if each team member knows where their place is and what they will be doing.”

I:9 “I would say that it never happens that the resuscitation is completed without us knowing that we have done everything we could.”

I:6 “Yes, resuscitation still presents a professional challenge for me. There are very few remedial exams retakes.”

#### 3.1.3. Education

Regarding the third topic, we found that all interviewees emphasized the importance of continuous professional education in resuscitation. They also underlined the importance of monitoring guideline changes because of new professional findings. The majority rated their knowledge of the guidelines for both TPO and DPO as good. The most significant shortcoming was the knowledge of therapy use in children.

Characteristic quotes: 

I:7 “I know what I’m doing professionally and stand by it.”

I:3 “Knowledge is always insufficient!”

#### 3.1.4. Reflection

Regarding the fourth theme, the interviewees often reflect on resuscitation interventions. They often perform the so-called self-reflection, where they mentally go through the intervention and determine its course and their role in it. Sometimes, they do it on purpose, but other times, the intervention remains in their minds. This type of thinking is considered good, as it is often aimed at studying possible mistakes and opens up possibilities for improvements. The most common form of reflection is a conversation with colleagues and team members who performed the resuscitation together. It usually takes place in an informal shape. It can start while driving from the intervention, when cleaning the workspace, the ambulance, restocking the used material, etc. Often, the intervention comes up during a coffee break. The respondents highlighted the importance of meetings after resuscitation (debriefings).

Characteristic quotes: 

I:8 “However, we can never avoid this question after resuscitation: What could be better or worse?”

I:10 “I wish post-resuscitation conversations among resuscitation team members were standard!”

I:11 ”Debriefing or open conversation immediately after the event which some are incapable of, but most can do.”

I:1 “After 20 years, I still perform self-analysis of resuscitations.”

I:6 “However, it would be welcome to include an additional expert, e.g., a psychologist.”

#### 3.1.5. Stressful Situations

In the fifth theme, one of the interviewees stated that resuscitation does not cause him “more” stress than other interventions. The rest, however, indicated a greater or lesser stress level, depending on the type of resuscitation. The resuscitations of children, resuscitations when faced with at least a partial lack of knowledge, and resuscitations when things became complicated were still described as the most stressful. Resuscitations when things did not go as they should have were also described as disturbing events (e.g., establishing venous access, an endotracheal tube, etc.). Particularly, stress is presented when making decisions related to resuscitation (start or stop resuscitation, how long to resuscitate, which procedures to perform, etc.). Good professional knowledge, experience, and adequate self-confidence can significantly reduce the stress of resuscitation. The anticipation and prepreparation can also reduce stress (if we have sufficient information about the condition before the cardiac arrest or the event itself).

Characteristic quotes: 

I:1 “I have experienced quite a few threats from people (relatives, friends, etc.) during resuscitation. But often, people don’t mean what they say. They are also in stress.”

I:2 “The more things went wrong, the greater the hardships.” “Conflicts during resuscitations? Of course, this happens.”

I:5 “Aggressive traits often associated with alcohol, malfunctioning equipment, finding a difficult location. I have already experienced physical violence. It was an unpleasant matter, and I felt threatened at the time. Verbal violence doesn’t bother me that much anymore.”

I:8 “Conflict with relatives who wanted their loved one to live for a long time, but our opinion was different, then the conflict quickly arises.”

I:11 “After a failed resuscitation, we have to express condolences, and that’s one of the hardest things we have to do.”

#### 3.1.6. Ethical Dilemmas

Regarding the sixth topic, we found that the mentioned ethical dilemmas have quite a few common denominators, and almost all the interviewees had similar opinions. Some such issues were the dilemma of starting CPR on a nonperspective person for the sole purpose of practicing CPR, resuscitation in nonperspective situations, prolonging an unpromising life or agony, disagreeing with a team member (especially the leader) and deciding to go into conflict with him, performing resuscitation only because of bystanders, etc. The research participants also expressed a dilemma regarding an individual’s pre-expressed will to abandon resuscitation (the matters are legally and formally still quite unclear in our country). All respondents, except one, approve of resuscitation for organ donation.

Characteristic quotes: 

I:10 “We have manikins for practice; it is unacceptable to do this on people.”

“When the time comes, I think allowing a person to leave with dignity is more humane.”

I:7 “If a team leader demanded something I strongly disagreed with, he had to do it himself; I didn’t.”

I:6 “I certainly respect the DNR (do not resuscitate) decisions. The only dilemmas are when we don’t have accurate data on resuscitations, things are not legally and formally regulated, and sometimes the opinion of relatives is different from ours.”

#### 3.1.7. Personal Satisfaction

The respondents felt the most excellent personal satisfaction when participating in successful resuscitations (the resuscitated person was discharged from the hospital without significant neurological impairments). They also felt personal happiness when they were thanked by the survivor’s relatives or even by those who had been resuscitated themselves. Despite an unsuccessful resuscitation, more interviewees indicated that they felt good knowing they had done their best at that moment.

Characteristic quotes: 

I:3 “Because for every intervention, when you went and gave it your best, that is, you did everything you knew how, then nothing burdened you.”

I:9 “When you resuscitated a person who regained consciousness during the intervention and could even talk. That was awesome!”

I:11 “When the person you resuscitated personally says “thank you for saving my life.” You can only wish for that to happen in this profession.”

I:2 “There’s a special satisfaction at the end of the year when you get a New Year’s card, and it says, ‘Thank you for saving my father, grandfather.”

#### 3.1.8. The Undetected Effects of CPR on the Practitioner

Regarding the eighth topic, we found that almost all interviewees allow for the possibility or believe that participating in resuscitations has some effects on them that they are unaware of. Most of them became aware of this fact after several years of work experience. As proof of the undetected effect of reanimations on them, the interviewees expose that they involuntarily return to certain reanimations in their minds, even after a long time.

Characteristic quotes: 

I:1 “We can revive a dead person. How could that fact not touch me?”

I:4 “ Possibly, I think so because I’m only human too.”

I:5 “Yes, basically, reanimation accompanies you all that day or even longer, you have it in your mind.”

I:7 “Perhaps there is a subconscious stress or even fear that I am not even aware of?”

I:8 “Yes, of course there is. No matter how we take it, even after so many years of working in this job, every resuscitation leaves a mark on you, whether you realize it or not.”

#### 3.1.9. Resuscitation According to the Age of the Person Being Resuscitated

In this topic, we found that the age of the reanimated person dramatically affects the resuscitation experience. Above all, we have two poles here: the resuscitation of children or younger people and, on the other hand, the resuscitation of older people. Death is inevitable in the last period of one’s life. Children still have their whole lives ahead of them, and their death is not expected. That is why all the interviewees mentioned the fact that resuscitating children presents the most challenging form of resuscitation for them, both mentally and professionally. The resuscitation of adults was described as reasonably routine work, yet no one defined it as such in children. The unsuccessful resuscitation of the elderly, especially those with known prior serious illnesses, in a way, represents the natural end of life for interviewees.

Characteristic quotes: 

I:10 “Certainly, this is a difference, resuscitating a child. From the point of view that he is at the beginning of his life’s journey and is not at all at the age when death is a natural event, as compared to an older age, when these things are also expected and often completely understandable—their death.”

I:3 “With the elderly, it is easier to accept that he has reached the end of his journey. This is significantly more difficult for children.”

#### 3.1.10. Resuscitation Efforts

In the tenth theme, we found that the interviewees described resuscitation as a physically very demanding intervention. The equipment required for the intervention has a considerable total weight (monitor/defibrillator, paramedic cases, resuscitation case, aspirator, ventilator, stretcher, mechanical devices for chest compression, etc.). Suppose resuscitation is carried out in a prehospital environment. In that case, transferring all of the above to a particular floor of a building, hill, grove, forest, and the like is often necessary. During the resuscitation, the procedures must be carried out as quickly as possible, which also causes physical and mental stress. Basic resuscitation and correct chest compressions with the appropriate depth and frequency require heavier physical effort too. At the same time, the interviewees noted that the population is gaining weight, and the resuscitation of an obese person is physically more demanding. The interviewees associate mental stress with specific stressful events (e.g., resuscitation of children, injured persons, dangerous circumstances, etc.), conflict situations within the team or the environment, and complications during resuscitation. Furthermore, this includes ethical dilemmas and decision making. All but one interviewee stated that immediately after resuscitation, they would have some free time to relax physically and mentally.

Characteristic quotes: 

I:6 “There are physical stresses—first of all, getting to the place with all this equipment. CPR alone is a single major physical activity. And then, if it’s successful, you still have to get it all to the ambulance, which is often a huge burden.”

#### 3.1.11. Performing CPR According to the Environment

When experiencing resuscitation according to the implementation environment, the interviewees pointed out a significant difference depending on whether the resuscitation is performed in a hospital or prehospital setting. A resuscitation in a hospital environment represents a controlled environment and, as such, is less stressful for the resuscitators (temperature, light, height of the resuscitation table, etc.). Only medical professionals are usually present in the hospital environment. On the other hand, a prehospital environment is not controlled. One must resuscitate anywhere and in any condition (heat, cold, daylight, darkness, rain, snow, forest, grass, road, etc.). In the prehospital environment, relatives, friends, colleagues, random eyewitnesses, etc., may be present during resuscitations. They can be helpful or highly annoying. The prehospital environment can also be very dangerous for the prehospital team (traffic accidents, work accidents, violent events, fires, hazardous substances, presence of animals, etc.). This can cause additional stress or even delay or prevent the start of resuscitation. Another big problem is variously long access times or the time from the cardiac arrest until the beginning of resuscitation, which is, in most cases, also unknown. The aforementioned reduces the chances of a successful resuscitation and increases the chances of failure. In the field, however, we are severely limited in resources (personnel, equipment, additional options or help, insufficient information, etc.)

Characteristic quotes: 

I:7 “I think it’s mainly in support. We have limited staff in the prehospital environment; you can always find additional help in the hospital...”

I:8 “Prehospital treatment is more difficult. There are several reasons: difficult conditions, limited space, time, personnel, and material resources. In addition, you have this sudden event, and the situation may escalate further with relatives in one way or another.”

I:4 “They were playing football on the field, and he collapsed. We resuscitated him there for almost an hour. When we were ready to transport the patient, I looked around and saw his teammates kneeling in the grass and praying under the spotlights on the field.”

## 4. Discussion

Qualitative research methods can also give us the answers to ‘why’ and ‘how’ a phenomenon occurred, and not just those answering ‘what’, ‘where’, and ‘who’ [26]. We conducted interviews with 11 people and reached data saturation with this number. This means that one can no longer obtain new data through interviews, and there is no need for further data collection [27]. 

In nursing, we often explore the experiences of patients, their families, and nurses [25]. Nevertheless, using a thematic analysis of the interviews is rarely used [28,29]. For this reason, we decided to include this approach in our research to gain a deeper insight into the experience of those participating in resuscitation. 

In the interviews, the interviewees described many situations that certainly affected their psychological and physical state because they participated in resuscitation procedures. All the respondents pointed out the resuscitation of a child and the resuscitation of a familiar person as very stressful situations. One interviewee stated that the stress during the resuscitation of children is 20 to 30 times greater than during the resuscitation of an older person. Many other authors also note that the unsuccessful resuscitation of children is one of the most stressful situations in emergency medicine [30,31,32,33,34]. 

Participation, especially in failed resuscitations, results in a certain stress level for nurses. It comes from an inner belief that a good nurse should be successful in their work [13]. Kirchhoff and Beckstrand [35] also note that the death of a patient can constitute a kind of stress for the nurse, arising from the feeling of helplessness at the death of the patient. Participating in an unsuccessful resuscitation might also be followed by activating protective mechanisms. At the same time, the occurrence of PTSD is also possible [36]. After unsuccessful resuscitations, it is humane to offer condolences to relatives. As one of the interviewees stated, this is the most challenging part of an unsuccessful resuscitation. Expressing condolences to the parents of a deceased child causes a tremendous psychological burden. Therefore, it is necessary to have additional knowledge and skills and consider the specificity of the situation [37,38]. Human life must end at one point, and it most often happens naturally during the period of old age. Hence, the interviewees view the death of an older person as the last part of human life and do not experience their unsuccessful resuscitation as very stressful [39,40,41]. The presence of relatives during the resuscitation of children is significant [42]. We found the same standpoint among our interviewees.

The research conducted by Rafiei et al. [43] shows that the attitude towards the presence of relatives significantly correlates with a nurse’s self-confidence. The more self-confident nurses are, the more positive their attitudes towards the presence of relatives during resuscitation. Under certain conditions, the interviewees advocated for the presence of relatives during resuscitations. However, this can burden the resuscitation teams as they have to deal with the emotions of those present. Many authors note that the presence of relatives during resuscitation can facilitate the acceptance of death and mourning. At the same time, they note that the presence of relatives did not affect the quality of the resuscitation team’s work. 

Regarding the unconscious effect of resuscitation on the interviewees, they all believe this effect may exist. One of the interviewees, who no longer works in the emergency department, stated that he had been unaware of this before. However, looking back now, he is convinced that participating in resuscitation procedures had some influence on him that he did not consciously perceive at the time. Healthcare workers sometimes decide on certain interventions or decisions based on their past experiences, and they may or may not be aware of them [44,45]. The effects of participation in resuscitation procedures on the emotions of healthcare providers have also been determined by many researchers, who have found that the participation in resuscitation procedures has a certain impact on providers’ feelings. This impact also depends on the type and outcome of the resuscitation. Research shows that most healthcare workers successfully compensate for the stress that comes with it [9,12,36,46,47,48,49,50], and the same was established in our research. The respondents know how to cope with the stress resulting from participating in resuscitation procedures. However, this should not mislead us into not recognizing the problem and the need for a personal and systemic institutional approach to solving the problem, which is certainly present to some extent. 

Surprisingly, the interviewees cited a somewhat burdensome situation when the equipment was inactive or not brought to the scene. Checking the equipment operation, delivery to vehicles, and transport to events is the rescuer’s domain. Therefore, it is understandable that they also pointed out irregularities in this area, since nonfunctioning equipment or its absence can affect the resuscitation outcome. Some other authors also state that every minute of delay in measures due to inadequate equipment or its lack of can reduce the chance of a successful resuscitation. At the same time, they emphasize the importance of situational awareness, which means being aware of the event and the surroundings [51,52,53,54]. 

The interviewees also highlighted the importance of good professional knowledge (guidelines for resuscitation), which reduces stress with appropriate experience and self-confidence. They also saw opportunities for improvement in the mentioned area. 

Furthermore, the interviewees pointed out the formal and legal vagueness of the procedures that the interviewees can carry out in the absence of a doctor. The proper legal regulation of jurisdiction is a matter of each country, and in Slovenia, these powers are not yet regulated satisfactorily [55]. The interviewees want more authority and additional knowledge in several areas. They are also aware that greater powers would bring an additional responsibility. 

In the interviews, we found dysfunctional relationships within the resuscitation team as a disturbing factor when the interviewees disagreed with the decisions of the resuscitation team leader or a colleague. In some cases, they described the mentioned situation as quite unpleasant for them. In their research in Finland, Azimirad et al. [56] found that more than half (64%) of the interviewed nurses believe they are not included in decision making as resuscitation team members. Half of them (50%) believe that their contribution to teamwork is not recognized. A common finding of the interview analysis in our research is that the interviewees often did not dare to defend their positions at the beginning of their professional journey. Now, with more knowledge, experience, and, as some have stated, authority, they stand more firmly behind their views. Their opinion is that resuscitation is a team effort, and thus, decisions must also result from some consensus among the team members. Considering the professional work and possible mistakes in this regard, all the interviewees advocated for team discussions after resuscitation, or “debriefing”. Many other authors also note the importance of regular debriefings after resuscitation. They believe there should be an open conversation, and the mistakes made from a purely professional point of view should be pointed out with the purpose of improving the work in the future. Such discussions enhance the quality of further work, improve relationships between colleagues, and help to deal with unpleasant feelings of team members [14,30,33,34,57,58,59]. During stressful work in emergency medical care, medical staff can also be exposed to the so-called second victim phenomenon. The phenomenon highlighted two types of victims in adverse events within healthcare settings. One victim is a patient or his family, and another is a healthcare worker involved in the event. This phenomenon is usually caused by errors that occur to health workers during their work [60]. After a systematic review of the literature, it was found that the prevalence of second victims after an adverse event varied from 10.4% up to 43.3%. The coping strategies used by second victims impact their patients, colleagues, and themselves. Women report more significant personal distress and are more willing to correct and learn from mistakes [61]. The consequences for healthcare workers can also be severe: insomnia, nightmares, reliving the incident repeatedly, loss of trust by their colleagues, lack of self-confidence, and fear of making another error; second victims blame themselves or feel ashamed of their responses to the clinical event, second-guessing their clinical skills and knowledge and feeling personally responsible for the unexpected patient outcomes [60,62]. When an adverse event occurs, support networks must be in place to protect the patient and the healthcare providers involved. Healthcare leaders must be aware of the high prevalence of second victims within their organizations and provide supportive interventions in the aftermath of adverse events [61].

The interviewees presented the participation in successful resuscitations with minimal neurological impairments of the revived person as the most encouraging. As examples, they cited the personal gratitude expressed by the resuscitated individuals, which has given them an extraordinary additional impetus for further work. Such situations provide very positive effects from participation in resuscitations.

There are some limitations in the research: We conducted interviews only among nurses working in the prehospital environment. In the survey, we wanted to cover persons with extensive resuscitation experience with or without a physician as part of a resuscitation team. We included only male respondents in the survey, as they represent the majority of employed nurses working in the prehospital environment in Slovenia. We also note that it does not make sense to generalize (inferential generalization) the obtained data to the entire population of nurses in Slovenia, since we surveyed a specific professional population (prehospital nurses and only the male population). However, we believe the obtained data could be generalized (representational generalization) for the mentioned population.

## 5. Conclusions

Regarding their experience in reanimation, many respondents mentioned that they are also “only human” and that reanimation touches them. The interviewees stated that sometimes they think about previous resuscitations, because some “can’t be forgotten”. The interviewees often stated that they feared resuscitation at the beginning of their professional careers. This feeling has diminished with experience; however, they still have much respect towards resuscitation. Resuscitation still constitutes a professional challenge for the interviewees, as no such intervention is like the previous ones. One can conclude that, according to the analysis of the interviews, the interviewees believe that due to stressful situations at the workplace (including resuscitation), they have learned to react decisively, collectedly, quickly, and professionally. This kind of experience, which they have somehow internalized, has also often proved useful in their private lives. The qualities listed above are necessary for working in emergency medicine, and over time, they certainly—at least partially—also shape a person.

## Figures and Tables

**Table 1:** Themes and semantics patterns.

No.	Theme	Semantics Patterns: (The Numbers Define the Semantic Patterns Defined Above)
1.	Effects of resuscitation on performers’ emotions	1, 2, 3, 6, 9, 10, 11, 12, 13, 15, 19, 20, 21, 23, 24, 26, 27, 29, 30, 33, 35
2.	Professional attitude	2, 3, 6, 10, 17, 29, 30, 31, 32, 34, 35, 36
3.	Education	4, 5, 17, 19, 30, 31, 34, 36
4.	Reflection	2, 3, 6, 7, 11, 14, 17, 24, 27, 31
5.	Stressful situation	2, 3, 8, 10, 12, 19, 25, 26, 29, 30, 31, 33, 35
6.	Ethical dilemmas	14, 17, 19, 20, 21, 30, 31,
7.	Personal satisfaction	14, 15, 17, 23, 27, 31, 32
8.	The undetected effects of CPR on the practitioner	12, 18, 19, 23, 26, 27, 33
9.	Resuscitation according to the age of the person being resuscitated	26, 30, 22a, 22b
10.	Resuscitation efforts	25, 26, 29, 30,
11.	Performing CPR according to the environment	28, 29, 30

Note: The theme is defined by a larger number of semantic patterns, and individual semantic patterns participate in the formation of several themes.

## Data Availability

Data supporting the findings of this study are available upon request from the corresponding author.
